# Current State and Future Perspectives on Gastroretentive Drug Delivery Systems

**DOI:** 10.3390/pharmaceutics11040193

**Published:** 2019-04-20

**Authors:** Julu Tripathi, Prakash Thapa, Ravi Maharjan, Seong Hoon Jeong

**Affiliations:** College of Pharmacy, Dongguk University-Seoul, 32 Donggukro, Ilsandonggu, Goyang, Gyeonggi 10326, Korea; julutripathi@gmail.com (J.T.); thapap139@gmail.com (P.T.); mrisnar@gmail.com (R.M.)

**Keywords:** gastroretentive drug delivery systems, gastric retention time, narrow absorption window, bioavailability, polymer

## Abstract

In recent years, many attempts have been made to enhance the drug bioavailability and therapeutic effectiveness of oral dosage forms. In this context, various gastroretentive drug delivery systems (GRDDS) have been used to improve the therapeutic efficacy of drugs that have a narrow absorption window, are unstable at alkaline pH, are soluble in acidic conditions, and are active locally in the stomach. In this review, we discuss the physiological state of the stomach and various factors that affect GRDDS. Recently applied gastrointestinal technologies such as expandable, superporous hydrogel; bio/mucoadhesive, magnetic, ion-exchange resin; and low- and high-density-systems have also been examined along with their merits and demerits. The significance of in vitro and in vivo evaluation parameters of various GRDDS is summarized along with their applications. Moreover, future perspectives on this technology are discussed to minimize the gastric emptying rate in both the fasted and fed states. Overall, this review may inform and guide formulation scientists in designing the GRDDS.

## 1. Introduction

Oral drug delivery systems have dominated other drug delivery systems for human administration due to their various advantages including ease of administration, flexibility in formulation, cost-effectiveness, easy storage and transport, and high patient compliance. However, oral drug delivery systems face challenges such as low bioavailability due to the heterogeneity of the gastrointestinal system, pH of the commensal flora, gastric retention time of the dosage form, surface area, and enzymatic activity [1]. Conventional drug delivery systems may not overcome the issues imposed by the gastrointestinal tract (GIT) such as incomplete release of drugs, decrease in dose effectiveness, and frequent dose requirement. Therefore, the failure of conventional drug delivery systems to retain drugs in the stomach may lead to the development of GRDDS. These systems offer several benefits such as prolonged gastric residence time (GRT) of dosage forms in the stomach up to several hours, increased therapeutic efficacy of drugs by improving drug absorption, and suitability for targeted delivery in the stomach. In addition, GRDDS can enhance the controlled delivery of drugs by continuously releasing the drug for an extended period at the desired rate and to the desired absorption site until the drug is completely released from the dosage form [1,2].

GRDDS are feasible for drugs that have low absorption in the lower part of the GIT, are unstable and poorly soluble at alkaline pH, have a short half-life, and show local activity at the upper part of the intestine for eradication of *Helicobacter pylori* [3,4,5,6,7,8,9,10,11,12,13,14,15]. Several formulation strategies have been used to design successful controlled release GRDDS including superporous hydrogel, bio/mucoadhesive, raft-forming, magnetic, ion-exchange, expandable, and low- and high-density systems [3,4,5,6,7,8,9].

Various formulation-related factors such as polymer types (nonionic, cationic, and anionic polymers), polymer composition in dosage form, viscosity grade, molecular weight of the polymer, and drug solubility can affect the quality of the gastroretentive dosage form [9]. Moreover, the physicochemical nature of excipients plays an important role in various GRDDS. For instance, density of excipients and composition of effervescent agents are critical factors in effervescent floating systems. In the case of superporous hydrogel systems, high swelling excipients such as crospovidone and sodium carboxymethylcellulose are required to form a superporous hydrogel [9,16]. Likewise, process variables can also influence the quality of the gastroretentive dosage form, as the density of a tablet can be altered by the compression pressure during tableting [9].

The main purpose of this review is to provide information on various GRDDS that have been developed to date, as well as the physiological state of the stomach, suitable drug candidates for GRDDS, factors affecting GRDDS, and in vitro and in vivo characterization of GRDDS. In addition, challenges and future perspectives on GRDDS are discussed.

## 2. Physiology of Stomach

**Figure 1:**
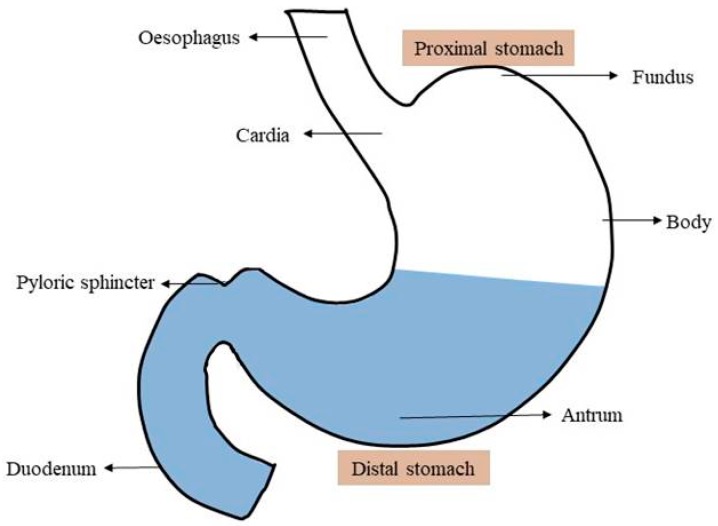
Schematic view on the anatomy of stomach.

In the GRDDS, the stomach has a crucial role; therefore, a good understanding of the anatomy and physiology of the stomach is a prerequisite for successful development of the gastroretentive dosage form. Anatomically, the stomach is divided into two parts: the proximal stomach, which consists of the fundus and body; and the distal stomach, which consists of the antrum and the pylorus as shown in Figure 1. The main role of the stomach is to store the food temporarily, grind it, and then slowly release it into the duodenum [17]. The fundus and body primarily act as reservoirs for undigested food, whereas the antrum acts as a pump to assist in gastric emptying by a propelling action [17,18]. The mobility pattern of the stomach is termed as the migrating myoelectric complex (MMC); the different phases of the MMC are presented in Table 1. Gastric emptying occurs in both the fed and fasted states, but the pattern of gastric emptying drastically varies between both states. In the fasted state, an interdigestive sequence of electrical events follows in a cyclic manner through both the stomach and the small intestine every 90–120 min [17]. During the interdigestive phase, the diameter of the pylorus increases up to approximately 19 mm [1,19]. As a result, particles smaller than the diameter of pyloric sphincter can easily evacuate from the pylorus to the duodenum during the interdigestive phase [19,20]. However, in the fed state, motor activity is generated 5–10 min after ingestion of a meal and continues as long as the food remains in the stomach, which can delay the gastric emptying rate.

## 3. Application of GRDDS

Suitable drug candidates for GRDDS are introduced in Table 2. Even though various types of GRDDS are reported in the literature, floating and mucoadhesive systems are the most popular gastroretentive dosage forms in pharmaceutical companies and contribute the most to the market. Table 3 presents the commercially available gastroretentive dosage forms.

## 4. Critical Factors Affecting GRDDS Efficacy

There are various factors that affect the performance of gastroretentive dosage forms. These factors are mainly categorized into pharmaceutical factors, physiological factors, and patient-related factors.

### 4.1. Pharmacutical Factors

For the successful design of GRDDS, it is important to understand the role of excipients and polymers on various types of GRDDS [9]. For instance, in the mucoadhesive system, polymers with high mucoadhesion strength, such as carbopol and hydroxypropyl methylcellulose (HPMC) may be required for successful design of the mucoadhesive dosage form. Likewise, with the expandable system, polymers with high swelling properties are more desirable. Moreover, the molecular weight, viscosity, and physiochemical properties of polymers can also affect the dosage form. Other formulation components such as gas generating agents in an effervescent floating tablet, high swelling excipients of sodium croscarmellose, and crospovidone for superporous hydrogels may be required. 

Moreover, the shape and size of the dosage unit is also important [38]. Garg and Sharma reported that ring shape and tetrahedron-shape dosage forms have a longer GRT compared to other shapes [39]. In most cases, the GRT of the dosage form is proportionately dependent on the size. An increase in the size of the dosage form could prevent its passage through the pyloric antrum in the intestine due to the size of the dosage form being larger than the pyloric sphincter diameter (mean, 12.8 ± 7 mm) [40]. Similarly, the density of the dosage form is also an important factor for low- and high-density systems. In low-density systems, the density of the dosage forms should be lower than that of the gastric fluid (1.004 g/cm^3^) in order to float in the gastric environment [41,42]. Increasing the floating capacity can improve the GRT of the low-density system; however, this effect is decreased in the presence of food. Moreover, the floating force of the dosage form decreases as a function of time, which could be due to the hydrodynamic equilibrium [43]. On the other hand, in high-density systems, the density of the dosage form should be greater than that of the gastric fluid so that it can sink in the bottom of the stomach and prevent gastric emptying. An increase in density of the dosage form greater than 2.500 g/cm^3^ enhances the GRT [44].

### 4.2. Physiological Factors

Several studies have reported that various extrinsic factors including the nature of meal, caloric content (caloric density and nature of the calories), frequency of ingestion, posture, sleep, and physical activity can affect the GRTs of drugs in the stomach [1,38,45,46]. In fasting states, gastrointestinal motility is represented by the MMC that occurs every 90–120 min [17]. During this period, motor activity sweeps undigested material from the stomach. If the timing of formulation administration coincides with that of the MMC, the GRT of the unit is very short. However, in the presence of food in the stomach, the MMC is interrupted and housekeeper waves are not generated leading to a prolonged GRT [17,47]. Likewise, the gastric emptying rate is also affected by the caloric density and nature of the calories of the ingested food [45]. In general, an increase in the caloric density significantly increases the GRT whereas the nature of calories only have a minor effect in the GRT [48]. In addition, high food viscosity may also increase the GRT [49,50]. Furthermore, the GRT is influenced by posture, and the effect is different for floating and non-floating dosage forms [1]. In the upright position, the floating system floats in the gastric fluid for a prolonged amount of time which can eventually increase the GRT. However, in similar conditions, the non-floating system remains in the lower part of the stomach and the gastric emptying rate is faster as a result of peristaltic contractions [1]. In contrast, in the supine position, the non-floating system has a longer GRT compared to the floating system [51,52].

### 4.3. Patient-Related Factors

Patient-related factors such as gender, age, illness, and emotional state can influence GRDDS. A recent study reported that gender affected the gastric emptying time and intraluminal pH [53]. The authors demonstrated that females had slower gastric emptying times than males [53]. Hormonal influences could explain the longer GRT in females than in males. Another study showed that males secreted more gastric acid compared to females [54]. Likewise, the age of the patient also affects the GRT. Elderly patients have a longer GRT compared to younger patients [55]. The nature of a patient’s illness may also affect the GRT of the dosage form. For instance, patients with Parkinson’s disease have a prolonged GRT that is frequently accompanied by constipation [56]. Likewise, in diabetic patients, gastric emptying is decreased by 30–50% [57]. The emotional condition of a patient may also influence GRDDS. It was reported that a decrease in gastric emptying rate was observed in patients suffering from depression, whereas an increased rate was observed in patients experiencing anxiety [38,47].

## 5. Current Pharmaceutical Technologies of GRDDS 

In this section, we describe currently used gastroretentive drug delivery approaches. The main mechanism of GRDDS includes floating, sinking, swelling, effervescence, mucoadhesion, and magnetic properties. A brief description for each system is summarized in Table 4.

### 5.1. Low-Density Systems

**Figure 2:**
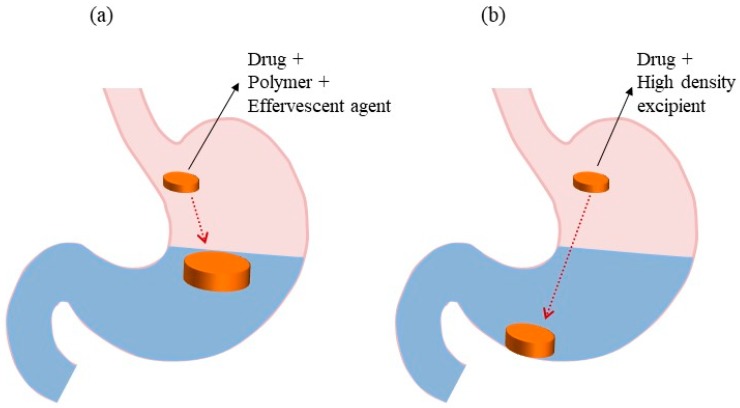
GRDDS based on (**a**) low-density systems and (**b**) high-density systems.

Low-density/floating systems are the most practical and extensively studied gastroretentive dosage forms [1,17,58,59]. The floating system was first introduced by Davis in 1968. In this system, the bulk density of the dosage form is lower than that of the gastric fluid (1.004 g/cm^3^). This property allows the system to remain buoyant in the stomach for a prolonged period of time while the drug is released at the desired rate from the system during the GRT [1,17,60]. Figure 2a illustrates the concept of low-density systems. These systems are classified into two subtypes based on the mechanism of buoyancy: non-effervescent floating and effervescent floating systems.

#### 5.1.1. Non-Effervescent Floating Systems

In non-effervescent systems, highly swellable cellulose derivatives or gel-forming polymers are used [5]. The formulation technique of non-effervescent systems involves mixing the drug with a gel-forming polymer. Various non-effervescent systems include the hydrodynamically balanced system (HBS), single- and double-layer floating tablets, and microballoons/hollow microspheres. 

The HBS system was first designed by Sheth and Tossounian in 1984 [65]. It is a single unit dosage form composed of one or more gel-forming hydrophilic polymers. HPMC, hydroxy propyl cellulose (HPC), hydroxyethylcellulose, sodium carboxymethylcellulose, carrageenan, agar, and alginic acid are some of the polymers that are used to design the HBS system [17,66]. In this system, the drug is mixed with the polymer and filled in the gelatin capsule. 

The floating tablet can be designed by uniform mixing of the drug and gel-forming hydrophilic polymer, which hydrates and swells upon contact with the gastric fluid and maintains the bulk density of the tablet at <1 g/cm^3^ [17,67]. Thus, the low-density systems float on the gastric fluid and prolong the GRT. The commonly used hydrophilic polymers in floating tablet include HPMC, polyethylene oxide, HPC, and cellulose acetate phthalate. Wei et al. [34] studied the bilayer floating tablet containing an immediate-release layer and sustained-release layer of the drug. The immediate-release layer contained a disintegrating agent, which aided the prompt release of drug, whereas the sustained release layer contained a hydrophilic polymer to control the drug release rate and also provided the tablet buoyancy. Drug-loaded microballoons/hollow microspheres are formulated by simple solvent evaporation or solvent diffusion techniques [68], and are multiple-unit floating systems. Polycarbonate, cellulose acetate, calcium alginate, Eudragit S, agar, and low-methoxylated pectin are commonly used polymers to design microballons. Various formulation variables such as the amount of polymer, ratio of plasticizer and polymer, and solvent can affect the floating behavior and drug release of these kinds of dosage forms [69]. One drawback of the HBS is that this system, being a matrix formulation, consists of a blend of drug and low-density polymers. The release kinetics of the drug cannot be changed without changing the floating properties of the dosage form and vice versa.

#### 5.1.2. Effervescent Floating Systems

Effervescent floating systems include a gas-generating agent and volatile liquids. This approach has been applied for single- and multiple-unit systems. In the gas-generating floating system, effervescent agents such as sodium bicarbonate, calcium carbonate, tartaric acid, and citric acid are used in combination with hydrophilic polymers [9,70]. When this system comes into contact with gastric fluid, CO_2_ is liberated due to the reaction of the effervescent agent with gastric fluid. The liberated CO_2_ gas is entrapped in the hydrocolloid matrix, which provides the tablet buoyancy and influences the drug release properties [71]. In volatile liquid systems, volatile liquids such as ether and cyclopentane are introduced into an inflatable chamber, which volatilize at body temperature allowing inflation of the chamber in the stomach [38]. Hydrophilic polymers are often used to control the drug release rate in this system. 

Effervescent floating systems can be categorized into single- and double-layer effervescent floating tablets and multiple-unit effervescent floating systems [14,17]. Single-layer effervescent tablets are formulated by intimately mixing effervescent agent, polymer, drug, and excipients. However, in bilayer effervescent floating tablets, one layer comprises the drug, polymer, and CO_2_ gas-generating agent, whereas the other layer constitutes an immediate-release drug and excipients without CO_2_ and polymer. In a recent study, sodium bicarbonate in HPMC matrix formulation was used to improve the GRT by increasing the hydration volume of dosage form and increasing the surface area of drug diffusion [14]. In addition, an increase in the amount of sodium bicarbonate decreased the drug release rate from the matrix, which could be due to obstruction of the diffusion path by CO_2_ gas bubbles [14]. Another study also utilized this approach to evaluate the in vitro and in vivo behaviors of ciprofloxacin hydrochloride effervescent floating tablets [72]. The optimized formulation was selected based on the GRT in humans (i.e., 5.50 ± 0.77 h). Multiple-unit effervescent floating systems consist of sustained-release pills as seeds surrounded by double layers. The inner layer contains effervescent agents such as sodium bicarbonate, calcium carbonate, and tartaric acid whereas the outer layer consists of polymers with swelling properties [17]. A low density system may be associated with problems such as sticking together or being obstructed in the GIT, which could produce gastric irritation. This system requires high fluid levels in the stomach to float and work effectively. Therefore, drugs with irritant effects on the gastric mucosa are not suitable candidates for low-density systems [1,17,20,47].

### 5.2. High-Density Systems

High-density systems have a density greater than that of gastric fluid (Figure 2b). Commonly used excipients of these systems include barium sulfate, zinc oxide, iron powder, and titanium dioxide [17]. In 1930, Hoelzel first discovered the effects of dosage form density on the GRT of several animal species. The densities of the tested dosage forms ranged from 0.9 to 10.5 g/cm^3^. The author concluded that high-density materials had slower GRTs than light-density materials. Thereafter, the impact of dosage form density on GRT has been studied. Garg and Gupta [51] reported that small high-density pellets are able to resist gastric peristaltic movements due to their retention in the antrum rugae or folds, increasing the gastrointestinal tract time from 5.8 to 25 h. Even though this system has the potential to improve the GRT, it is difficult to design high-density pellets containing high-dose drugs. Moreover, only a few clinical studies on high-density pellet formulations have been reported in the literature; as a result, the clinical significance of these systems is still questionable [73]. Therefore, future directions need to be focused on animal studies to investigate the clinical significance of such dosage forms.

### 5.3. Expandable Systems

**Figure 3:**
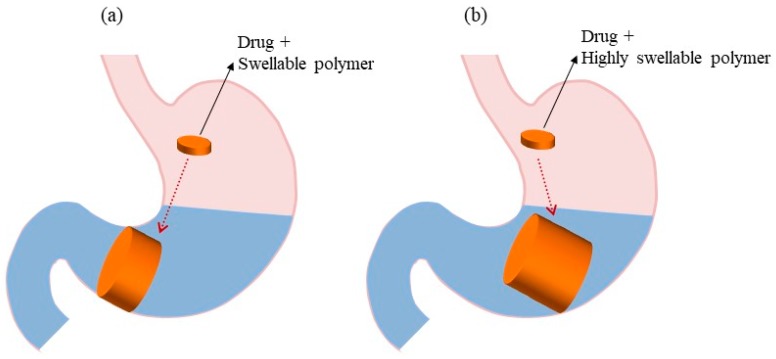
GRDDS based on (**a**) expandable systems and (**b**) superporous hydrogel systems.

Expandable drug delivery systems are designed to have a longer GRT through an increase in their volume or shape (Figure 3a). Initially, they were used for veterinary purposes and, subsequently, their applications were extended to humans [51]. Three general configurations need to be considered for the proper functioning of the system: small size for easy oral intake, expanded form in the stomach to prevent passage through the pyloric sphincter, and size reduction of the system after complete drug release to enable evacuation [1,6,12]. This system is also termed as a “plug type system” because it has the ability to block the pyloric sphincter. Expansion of the system occurs by two methods, swelling and unfolding, which allow for volume and shape modification, respectively [6,18]. The main mechanism for swelling and drug release from the system is diffusion. These systems utilize hydrophilic polymers (e.g., HPMC, polyethylene oxide, and carbopol) that can absorb water from the gastric fluids and increase the volume of the system. Likewise, in unfolding systems, the polymer and drug are in a folded/compressed state inside the gelatin capsule. When they come into contact with the gastric fluid, gelatin is dissolved and releases the mechanically preferred expanded configuration. Different geometrical forms of biodegradable polymer can be prepared and compressed within a capsule [12]. It is crucial to select a suitable biodegradable polymer with an appropriate molecular weight, viscosity grade, and swelling properties to maintain the sustained release profile of the dosage form [12,74]. Various novel polymers have the ability to swell promptly in contact with the GI fluid. Sivaneswari et al. developed and characterized a novel expandable GRDDS of levetiracetam based on an unfolding mechanism. In their study, the drug was loaded onto a polymeric patch made of HPMC, carbopol 934 P, and xanthum gum, which was designed to adhere to the gastric mucosa, where the drug was released in a sustained manner. Chen et al. [61] formulated GRDDS tablets of Losartan using an equivalent ratio of hydroxyethylcellulose and chitosan, and sodium bicarbonate was added as a gas-generating agent. The optimized formulation showed a sustained release profile of more than 16 h with good swelling and floating behavior. Their results suggested that the addition of sodium bicarbonate could improve the floating ability of the dosage form but might reduce the swelling ability of the low viscosity grade of chitosan. Therefore, the optimum ratio of the polymer and gas-generating agent is necessary to obtain the preferred GRDDS. However, expandable systems have a few limitations such as difficulty in storing easily hydrolysable, biodegradable polymers; being difficult to manufacture and may not be cost-effective; difficulty in maintaining the structural integrity; and may cause bowel obstruction, intestinal adhesion, and gastropathy [6,12,61].

### 5.4. Superporous Hydrogel Systems

In 1998, the superporous hydrogel was presented as a different category of water-absorbent polymer system. This system has gained popularity in the controlled-release formulation due to its high mechanical strength and elastic properties [75]. It has a pore size greater than 100 μm, and as a result, it swells rapidly to an equilibrium size due to water uptake by capillary wetting through numerous pores. Figure 3b depicts the schematic concept of the superporous hydrogel system. The conventional hydrogel system is a slow process and takes several hours to reach equilibrium; thus, the dosage form can be easily evacuated from the stomach. On the contrary, the superporous hydrogel systems swell up to 100 times or more, and gain enough mechanical strength to withstand pressure by gastric contraction, thereby increasing the GRT. Highly swellable polymers, such as croscarmellose sodium and sodium alginate are used in these systems [12,64]. However, these systems can be highly sensitive to pH, and swelling can be reversible due to changes in pH and poor mechanical strength of the structure [12,64].

### 5.5. Bioadhesive/Mucoadhesive Systems

**Figure 4:**
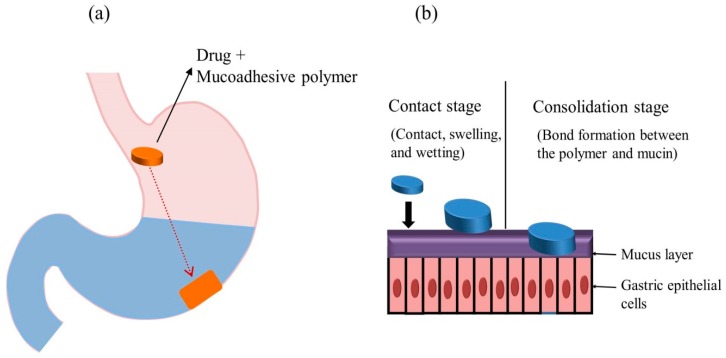
Mucoadhesive GRDDS (**a**) general representation of mucoadhesive systems and (**b**) mechanism of mucoadhesive systems.

The mucoadhesive/bioadhesive system was first introduced by Park and Robinson in 1984 [76]. It was designed to adhere to the gastric epithelial cell surface and prolong the GRT of drug compounds [8,17]. Figure 4a illustrates the concept of this system. In this approach, drugs are incorporated in a mucoadhesive agent, which can be either natural or synthetic polymers. Bonding established between the polymer and mucosal surface facilitates the mucoadhesion process [77], which generally involves two steps: the contact stage and the consolidation stage (Figure 4b) [78]. The mechanism of mucoadhesion is highly complex and is not fully understood; however, different theories have been postulated, as summarized in Table 5. Various gastrointestinal mucoadhesive dosage forms such as beads, microspheres, films, capsules, and tablets have been prepared and reported in the literature [79]. Commonly used mucoadhesive polymers include carbopol, chitosan, sodium alginate, HPMC, polyethylene glycol, and poly(acrylic acid) [12,17]. Mucoadhesive polymers assist in binding drug substances to the mucosal surfaces and prolonging the drug residence time at the application site. An ideal mucoadhesive polymer is inert, non-irritating, nontoxic, adheres to the mucosal surface, and possesses site specificity; and interacts with the mucin through electrostatic, disulfide, hydrogen, and hydrophobic bonding. The mucoadhesive properties and interaction strength of the polymer depend on the molecular weight, structure, flexibility of the polymeric chains, hydrogen bonding capacity, cross-linking density, charge, concentration, or hydration degree of the polymer [80]. 

Various studies have focused on the combination of floating and mucoadhesive properties in order to improve the GRT of the dosage form by forming mucoadhesive floating drug delivery systems (MFDDS) [61,80,82,83,84]. Liu et al. developed a hollow-bioadhesive microsphere of psoralen using glycerol monooleate as a bioadhesive polymer. The prepared microspheres showed strong mucoadhesive properties with good buoyancy both in vitro and in vivo. Moreover, the pharmacokinetic analysis of the drug in the microsphere showed the prolonged elimination half-life time and reduction in elimination rate. Recently, a novel gastroretentive oil entrapped alginate beads containing resperidone was prepared by using ionotropic emulsion gelation technique. The alginate beads were coated with the cross-linked alginate sterculia gum gel membrane. The dosage form demonstrated good floating and ex vivo mucoadhesion behavior and sustained drug release characteristics [84]. Generally, different types of ex vivo mucosal tissues are used to evaluate the mucoadhesive properties (total work of mucoadhesion, force of system detachment, percentage of mucoadhesion) of the dosage form [85,86,87]. However, mucosal tissues taken from laboratory animals can be difficult to obtain and are not considered good from an ethical point of view. In such cases, synthetic hydrogels that can mimic biological mucosa could be a good synthetic substitute for animal tissues. The hydrophilic structure of the hydrogels renders them capable of imbibing large amounts of water in their three-dimensional networks and resembling the properties of biological tissues [88]. In this system, specific targeting might be difficult because composition of the mucus is different according to the region of the mucous membrane. In addition, the constant turnover of the mucus, and high stomach hydration might decrease the bioadhesion of polymers. Moreover, there is high risk of adhesion to the esophagus which may lead to collateral lesions [12,45].

### 5.6. Raft-Forming Systems

**Figure 5:**
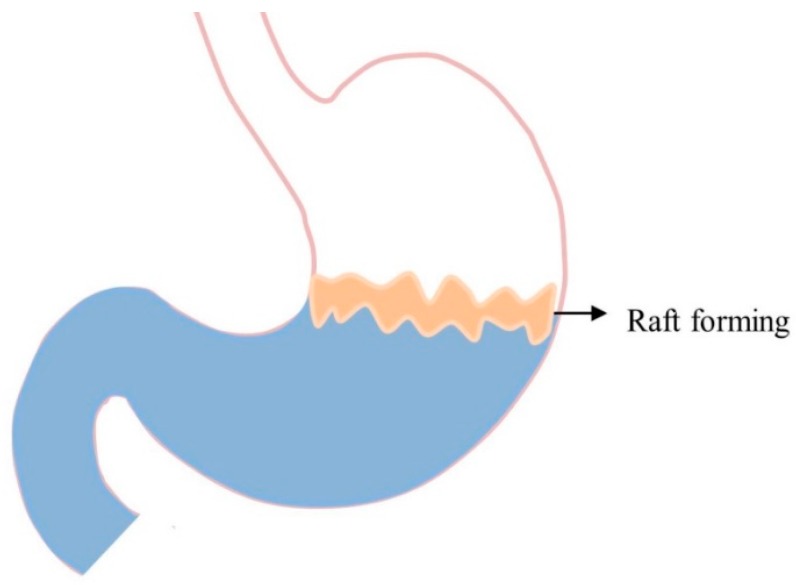
GRDDS based on raft-forming systems.

Raft-forming systems are another type of GRDDS, formulated with effervescent excipients and gel forming polymers in order to achieve the sustained drug delivery. Figure 5 illustrates the concept of these systems, which mainly focuses on achieving localized effects because floating rafts act as blockades between esophagus and stomach. Thus, they can be used for the effective management of gastric esophageal reflux disease. When raft-forming systems come into contact with gastric fluid, they swell and form a viscous cohesive gel leading to the formation of a continuous layer termed as rafts [17,62]. Fabregas et al. [89] explained the antacid raft-forming floating system. The authors used sodium alginate as a gel-forming polymer, and sodium bicarbonate and acid neutralizer as gas-generating agents. Thus, CO_2_ gas is generated that lowers the bulk density of the system, and as a result, the raft floats on the gastric fluid. Nabarawi et al. [62] developed a controlled release floating raft system of mebeverine hydrochloride, and evaluated different excipients for their floating behavior and in vitro controlled-release. It forms a viscous and cohesive gel when it swells and entraps CO_2_ bubbles produced by the reaction of carbonates and gastric fluid [12]. The formed raft can remain intact in the stomach for several hours, promoting the sustained release of the drug. Such rafts are particularly useful for delivering antacid drugs such as aluminum hydroxide, calcium carbonate, and simethicone [90]. However, the mechanical strength of the systems is weak and can be easily disrupted by the MMC [62,63].

### 5.7. Magnetic Systems

**Figure 6:**
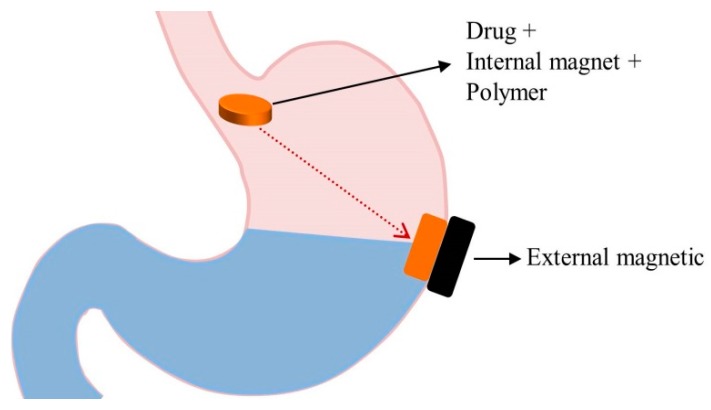
GRDDS based on magnetic systems.

In magnetic systems, a dosage form consists of active pharmaceutical ingredient, excipients and also a small amount of internal magnet. An extracorporeal magnet is placed over the stomach to control the position of the dosage form containing internal magnet as presented in Figure 6 [17]. The position and the magnetic field intensity of the extracorporeal magnet can affect the GRT [16]. Previous studies have reported that the GRT and bioavailability are improved by magnetic tablets [3,91]. Groning et al. performed a study in human volunteers using magnetic acyclovir tablets with and without an external magnetic field. The authors observed that the GRT and plasma drug concentration were increased in the presence of an extracorporeal magnet. Ito et al. formulated bioadhesive granules containing ultra-fine ferrite and performed in vivo experiment in rabbits [92]. They found that an external magnetic field intensity of 1700 G retained all granules in the stomach for more than 2 h. However, specific positioning of the magnet might be difficult and results in low patient compliance [16]. Only a few studies have been conducted on magnetic systems and their clinical significance has yet to be explored. Therefore, future research studies on these systems need to focus more on their clinical significance. 

### 5.8. Ion-Exchange Resin Systems

The ion-exchange resin system consists of the water insoluble cross-linked polymer (resin) that can be either cationic or anionic. In general, it is designed to release the drug in a controlled manner. The suitable resins can be chosen according to the drug properties. In case of GRDDS, drugs should be released in the stomach and hence this system is applicable to cationic drugs. Therefore, cationic resin can be selected. A specific amount of resin is poured on a known drug concentration and mixed homogeneously for a certain period. The drug ions from the solution get adsorbed onto the resin matrix and displace cations from the resin. Such loaded drug resin complexes are called resinates. When the resinates come into contact with the hydrogen ions in the acidic environment of the stomach, hydrogen ions are exchanged with the drug ions present in the resinates matrix. As a consequence, the drug ions are released into the gastric fluid while the resin particles are eliminated through the large intestine [93]. The release rate of the drug from resins depends on inherent properties of the resins such as the particle size, cross-linking density, type of ionogenic group. Moreover, it also depends on the nature of the drugs, ionic environment and test solution [93]. When an ion exchange resin is highly cross-linked, the drug loading efficiency gets decreased [94,95]. The degree of drug resin complexation can be calculated by dry weight resin capacity measurement methods. It is determined by weighing a dry resin, rewetting it in drug solution, and displacing completely from the resin. The displaced ions can be assayed giving the degree of drug resin complexation. Even though this system alone may not be suitable to increase the GRT, the ion exchange resin can be combined with floating delivery systems or bioadhesive systems to prolong the GRT [96]. Some of its limitations may be difficulty in estimating the amount of bound resin with drug, and safety issues concerning its ingestion.

## 6. Evaluation Parameters of GRDDS

### 6.1. In Vitro Evaluation Parameters

In vitro assessments of GRDDS can be used to predict the in vivo performance. The routine evaluation methods of gastroretentive tablets include measurement of tablet tensile strength, weight variation, friability, drug content, content uniformity, and in vitro drug release. Floating behaviors such as floating lag time and total floating duration have been used for the assessment of floating behavior of low-density systems. Furthermore, floating force is also used to measure the floating capacity of the floating tablet. In addition, swelling rate, water uptake capacity, and gel strength of the polymeric dosage form can be evaluated using dissolution medium and tested for at least 8 h to ensure the floating mechanism, drug release, and gel strength. Table 6 summarizes various in vitro evaluation parameters of different GRDDS. 

### 6.2. In Vivo Evaluation Parameters

In order to provide the evidence of in vivo efficacy of GRDDS, a well-designated in vivo study in an animal model or humans is required. In vivo studies provide information about the GRT and bioavailability of the drug. Selection of a suitable animal model is the first requirement for a successful in vivo study. For example, in small animals such as mouse, rat, guinea pig, and rabbit, there might be an issue of animal handling especially for large dosage forms [18,107]. As a result, measurements of the GRT and bioavailability are still difficult. Table 6 summarizes the in vivo evaluation parameters of different GRDDS. 

Various diagnostic imaging techniques including gamma scintigraphy, radiology, gastroscopy, ultrasonography, and magnetic resonance imaging (MRI) can be applied for in vivo evaluations of GRDDS [6,18,36,108]. Gamma scintigraphy studies have been conducted to determine the location and extent of GRDDS and their transit through the GIT. In this technique, small amounts of stable isotope are added to the dosage form during its preparation [18]. Then, this isotope is converted into γ-emitting material by irradiating the dosage form in a neutron source. Gamma rays are released and captured as an image after processing by a computer. This method can also be used for the identification of dissolution and disintegration properties of the dosage form. A good safety profile and relatively low doses of radiation are the major advantages of the technique [6,18].

Likewise, the radiology/X-ray technique is used for the preclinical evaluation of GRT, disintegration rate, dimensions of the dosage form, and esophageal transit of GRDDS [18,36]. In this technique, a radio-opaque material such as barium sulphate is incorporated with the dosage form, and radiographs taken after ingestion of the dosage form help in locating the dosage forms at various periodic time intervals. Its major advantages compared to γ-scintigraphy are simplicity and cost. Even though this technique has been successfully used in human volunteers, dogs, and rabbits, safety issues still need to be considered because repetitive exposure to x-rays may lead to various health hazards. 

Gastroscopy is a type of per-oral endoscopy used for the diagnosis and monitoring of GRDDS [18]. This technique composed of optical fibers and a video camera to determine the location of the dosage form. This method is applicable for all types of GRDDS; however, it is less convenient and might require minor or complete anesthesia to assess gastric retention of GRDDS. Similarly, ultrasonography is an alternative technique used in GRDDS. Ultrasonic waves are generated that enable the imaging of some abdominal organs and determine the intragastric location of the hydrogels, solvent penetration into the gel, and interactions between the dosage form and gastric mucosa during peristalsis [6]. MRI is another technique for determining the in vivo gastric retention of GRDDS. This technique uses magnetic fields and radiowaves to view the complete anatomical structure as well as location of the ingested dosage form [6,36]. The compounds with super paramagnetic properties (e.g., ferrous oxide) are incorporated for visualization purposes. Steingoetter et al. used this technique to report the in vivo gastric retention of gadolinium chelates floating tablets containing Fe_3_O_4_ as a super paramagnetic agent and succeeded in analyzing intra-gastric tablet position and residence time in human volunteers.

## 7. Future Perspectives of GRDDS

The GRT of the conventional dosage form is one of the main challenges in the pharmaceutical industry, especially for drugs that are absorbed from the upper part of the intestine. Developing GRDDS will help to overcome the drawbacks associated with conventional dosage form, although further work is needed on its shortcomings. To date, many studies have been performed on GRDDS utilizing the single system approach such as floating, expandable, and mucoadhesive systems. 

Even though various GRDDS technologies have been extensively explored to achieve successful gastroretentive systems, most have their own limitations (Table 4). The variation in GRT, especially in the fed and fasted states, is still one of the main challenges faced by many formulation scientists. No single approach might be the best for resolving the problems. Therefore, it is desirable to explore suitable GRDDS that can overcome the limitations of a single approach. Using combination approaches such as expandable and effervescent floating systems, mucoadhesive and floating systems, swellable and floating systems, and mucoadhesive and high-density system may be useful strategies for minimizing the variability of GRT. Moreover, dual-working systems are less affected by the physiological condition of the stomach such as the fasting and fed states and these systems can ensure delayed gastric emptying. Therefore, future works on GRDDS should be focused on combinations of different mechanisms in order to prolong gastric retention of dosage forms even in the fasted state. 

It is essential to assess gastroretentive dosage forms on a case-by-case basis because the physiochemical nature of drug and excipients, types and composition of polymers, drug dose, and manufacturability may depend on product specification [19]. Another important aspect for improving GRDDS is to understand the effects of formulation and process variables on the critical quality attributes of GRDDS. The critical quality attributes of GRDDS include floating behavior, floating force, gel strength, mucoadhesive strength, mucoadhesive time, in vitro drug release, swelling capacity, porosity of hydrogel, tablet tensile strength, and friability. From formulation viewpoints, understanding polymer behavior and its role in formulation is crucial for the rational development of the gastroretentive dosage form. Furthermore, selection of an appropriate concentration of polymer is equally important for designing such dosage forms. In this regard, the quality by design (QbD) approach can be a useful tool for investigating the influence of formulation and process variables on the critical quality attributes of GRDDS. With implementation of the QbD approach in pharmaceutical fields, there has been a significant transformation in the understanding and control of the manufacturing process, which notably minimizes the risk of product failure [9]. 

Some gastroretentive approaches such as magnetic systems have not been extensively studied. The clinical studies of these systems have not yet been reported in detail. Therefore, future works on magnetic systems need to be focused on clinical candidates to specify their practical applications in humans. Moreover, incorporating magnetic systems into the superporous hydrogel system can help extracorporeal magnets precisely locate the ingested dosage form since it swells and occupies larger volume. The advancement of technologies offers efficient measurement tools that can help to predict and correlate the gastric emptying time and passage of drug into the GIT. For example radiology and scintigraphy can be used for the *in vivo* evaluation of gastric emptying of dosage forms from the stomach [17]. Moreover, magnetic marker monitoring techniques can also be utilized to capture images of dosage forms in the stomach [109]. 

## 8. Conclusions

GRDDS have great potential to improve the therapeutic efficacy of drugs with narrow absorption windows, high solubility at acidic pH, and instability at alkaline pH. A thorough understanding of the anatomy and physiological state of the stomach, investigations into the impact of formulation and process variables on dosage form quality is a prerequisite for the successful design of GRDDS. Even though various GRDDS such as bio/mucoadhesive, magnetic, low-, and high-density systems have been reported in the literature, their clinical significance still needs to be studied. From the pharmaceutical aspect, future directions of GRDDS may need to focus on a combination approach of GRDDS to achieve better product quality. Moreover, a QbD approach can be used to better understand the effects of formulation and process variable on product performance.

## Figures and Tables

**Table 1:** Four phases of the migrating myoelectric complex [17,18].

Phase	Comments	Duration
Phase 1	Quiescent period with rare contractions.	30–60 min
Phase 2	Intermittent action potentials and contraction that gradually increases in intensity and frequency as the phase progresses.	20–40 min
Phase 3	Short periods of intense, large, regular contractions. This phase is termed as “housekeeper wave” as it enables all undigested materials to be swept out of the stomach and down to the small intestine.	10–20 min
Phase 4	Occurs between phase 3 and phase 1 of two consecutive cycles in a brief transitional phase.	0–5 min

**Table 2:** Suitable drug candidates for gastroretentive drug delivery systems (GRDDS).

Bioavailability Challenges	Drug	Therapeutic Indications	References
Local activity	Ranitidine, Amoxicillin, Levofloxacin, Metronidazole	Peptic ulcer and reflux esophagitis, eradication of *H. pylori*	[1,13,21,22,23,24]
Plasma fluctuations	Ciprofloxacin, Clarithromycin	Urinary tract, respiratory, and GI infections	[1,25,26,27,28]
Low solubility at alkaline pH	Ofloxacin	Urinary tract, respiratory, and GI infections	[1,10]
	Cinnarizine	Nausea, vertigo, and motion sickness	[15]
Narrow absorption window	Riboflavin	Essential nutrients, mouth ulcer and sore throat	[29,30]
	Cilostazol	Inhibits platelet aggregation	[4]
	Pregabalin	Fibromyalgia, diabetic peripheral neuropathy, post-herpetic neuralgia, and adjunctive therapy for partial onset seizures	[5,31]
Short half-life, narrow absorption window	Levodopa	Parkinson’s disease	[32]
	Metformin	Type II diabetes mellitus	[7,9,33,34]
Poor absorption from lower GIT	Atenolol	Hypertension	[1]
	Lafutidine	Gastric and duodenal ulcers	[35]
Unstable at alkaline pH	Verapamil, Captopril	Hypertension	[1,11,14]

**Table 3:** Various gastroretentive products available in the market [1,18,19,36,37].

Delivery Systems	Brand Name	Active Ingredient	Manufacturing Company
Bioadhesive tablets	Xifaxan^®^	Rifampicin	Lupin, India
Bilayer floating capsule	Cytotec^®^	Misoprostol	Pfizer, UK
Coated multi-layer & swelling system	Baclofen GRS^®^	Baclofen	Sun Pharma, India
Colloidal gel forming floating system	Conviron^®^	Ferrous sulphate	Ranbaxy, India
Effervescent floating system	Zanocin OD^®^	Ofloxacin	Ranbaxy, India
	Riomet OD^®^	Metformin hydrochloride	Ranbaxy, India
	Cifran OD^®^	Ciprofloxacin	Ranbaxy, India
Effervescent floating liquid alginate preparation	Liquid Gaviscon^®^	Alginic acid and sodium bicarbonate	Reckitt Benckiser Healthcare, UK
Effervescent and swelling based floating system	Prazopress XL^®^	Prazosin hydrochloride	Sun Pharma, Japan
Erodible matrix based system	Cipro XR^®^	Ciprofloxacin hydrochloride and betaine	Bayer, USA
Expandable system (unfolding)	Accordion Pill^®^	Carbidopa/levodopa	Intec Pharma, Israel
Raft forming system	Topalkan^®^	Aluminum magnesium	Pierre Fabre Medicament, France
	Almagate FlatCoat^®^	Aluminium-magnesium antacid	Pierre Fabre Medicament, France
Floating system—controlled release capsule	Madopar HBS^®^	Levodopa and benserzide	Roche, UK
	Prolopa HBS^®^	Levodopa and benserzide hydrochloride	Roche, UK
	Valrelease^®^	Diazepam	Roche, UK
Foam based floating system	Inon Ace Tables^®^	Simethicone	Sato Pharma, Japan
Gastroretention with osmotic system	Coreg CR^®^	Carvedilol	GlaxoSmithKline, UK
Minextab Floating^®^—floating and swelling system	Metformin HCl	Metformin hydrochloride	Galanix, France
	Cafeclor LP	Cefaclor	Galanix, France
	Tramadol LP	Tramadol	Galanix, France
Polymer based swelling technology: AcuForm^TM^	Gabapentin GR	Gabapentin	Depomed, USA
	proQuin XR	Ciprofloxacin	Depomed, USA
	Glumetza	Metformin hydrochloride	Depomed, USA
	Metfromin GR^TM^	Metformin hydrochloride	Depomed, USA

**Table 4:** Summarized mechanisms of the various GRDDS.

Gastroretentive Approach	Mechanism	References
Low-density systems/ floating systems	System causes buoyancy in gastric fluid. Density of pellets/tablets is lower than the density of stomach fluid.	[1,17,20,47]
High density systems	Uses the density of dosage form as a strategy to produce the retention mechanism. Sinking system remains at the bottom of the stomach, where the density of the dosage form is greater than the gastric fluid.	[1]
Expandable systems	Expansion of the dosage form occurs by swelling or unfolding in the stomach. Swelling usually occurs because of diffusion. Unfolding takes place due to mechanical shape memory.	[6,12,61]
Bioadhesive systems	A very complex process with several mechanisms, including electrical theory, adsorption, wetting, diffusion, and fracture theories. The interaction between the negatively charged mucosal surface and positively charged polymers might facilitate the bioadhesive process.	[12,45]
Raft forming systems	The polymer in presence of mono or di valent cations, absorbs water, swells and forms in situ gel layers, which float above gastric fluid and termed as raft.	[62,63]
Super-porous hydrogel systems	Swells up to 100 times due to water update by capillary wetting through numerous pores.	[12,64]
Magnetic systems	Consists of the small internal magnet mixed with the drug. Its position inside the stomach is controlled by an extracorporeal magnet.	[16]
Ion-exchange resin systems	Drug is loaded into the resin to form the resin loaded drug complex, which can be combined with floating delivery or bioadhesive systems.	[16]

**Table 5:** Suggested mechanisms of mucoadhesion [1,81].

Theories	Mechanisms of Mucoadhesion
Wettability	Bioadhesive polymers penetrate and develop intimate contact with the mucous layers.
Diffusion	Physical entanglement of mucin strands and flexible polymer chains. Influenced by molecular weight, cross-linking density, chain flexibility, and expansion capacity of both networks.
Adsorption	Bioadhesion is due to primary forces (ionic, covalent, and metallic) and secondary forces (van der Waals, hydrophobic and hydrogen bonds) between surfaces.
Electronic	Attractive electrostatic forces between the glycoprotein mucin network and the bioadhesive material.
Fracture	Detachment force needed to separate the mucus and polymer reflects the force of the adhesive binding.

**Table 6:** In vitro evaluation parameters of various GRDDS.

GRDDS	Evaluation	Comment	References
Low-density system, raft-forming system	Floating Lag Time (FLT), total floating time (TFT), floating strength	The test is carried out in a simulated gastric fluid (SGF) at 37 °C. The time between introduction of dosage form and its buoyancy on the SGF (FLT) and the time during which the dosage form remains buoyant (TFT) were measured. The floating strength is measured using specifically designed basket holder connected with analytical balance. The reduction of weight on the analytical balance over time determines the floating strength.	[9,17,19,31,97,98]
Superporous hydrogel system, expandable system	Swelling studies	The test is carried out by placing the weighed amount of dosage form into the swelling medium (0.01 N HCl) and weight, diameter, and length of swollen samples are measured at predetermined time point.	[61,99]
Raft-forming and Mucoadhesion systems	Viscosity and Rheology	Viscosity of polymer affects the consistency of the dosage form upon contact with the gastric fluid. Brookfield/Ostwald’s viscometer and texture analyzer are commonly used.	[100]
Expandable system	In vitro unfolding study	The test is carried out by placing the folded dosage form into the dissolution medium and examining its unfolding behavior in different time interval.	[101]
Ion-exchange resin system	Particle size, ion exchange capacity, moisture content	Particle size analysis is carried out using a sieve shaker, laser diffraction, and coulter counter analyzer. The ion exchange capacity depends upon the functional group available for crosslinking. Moisture content can be measured with Karl Fischer.	[96,102,103,104]
Applicable for all GRDDS	In vitro drug release	The test is carried out in SGF at a predefined time interval (generally 0 to 12 h) using USP type-II apparatus at 50 rpm and maintained at 37 °C.	[9,27,72,80]
	Gel strength	The high gel strength is desirable for better mechanical integrity.	[9,105]
	Drug-excipient interaction study	It can be studied by using FT-IR spectroscopy, Differential scanning calorimetry, and High Performance Liquid Chromatography.	[17,106]
